# Allosteric Inhibition of Neutral Sphingomyelinase 2 (nSMase2) by DPTIP: From Antiflaviviral Activity to Deciphering Its Binding Site through In Silico Studies and Experimental Validation

**DOI:** 10.3390/ijms232213935

**Published:** 2022-11-11

**Authors:** Hadrián Álvarez-Fernández, Patricia Mingo-Casas, Ana-Belén Blázquez, Flavia Caridi, Juan Carlos Saiz, María-Jesús Pérez-Pérez, Miguel A. Martín-Acebes, Eva-María Priego

**Affiliations:** 1 Instituto de Química Médica (IQM), CSIC, 28006 Madrid, Spain; 2 Department of Biotechnology, Instituto Nacional de Investigación y Tecnología Agraria y Alimentaria (INIA), CSIC, 28040 Madrid, Spain

**Keywords:** host-targeted antivirals, flavivirus, nSMase2, allosteric inhibition

## Abstract

*Flavivirus* comprises globally emerging and re-emerging pathogens such as Zika virus (ZIKV), Dengue virus (DENV), and West Nile virus (WNV), among others. Although some vaccines are available, there is an unmet medical need as no effective antiviral treatment has been approved for flaviviral infections. The development of host-directed antivirals (HDAs) targeting host factors that are essential for viral replication cycle offers the opportunity for the development of broad-spectrum antivirals. In the case of flaviviruses, recent studies have revealed that neutral sphingomyelinase 2, (nSMase2), involved in lipid metabolism, plays a key role in WNV and ZIKV infection. As a proof of concept, we have determined the antiviral activity of the non-competitive nSMase2 inhibitor DPTIP against WNV and ZIKV virus. DPTIP showed potent antiviral activity with EC_50_ values of 0.26 µM and 1.56 µM for WNV and ZIKV, respectively. In order to unravel the allosteric binding site of DPTIP in nSMase2 and the details of the interaction, computational studies have been carried out. These studies have revealed that DPTIP could block the DK switch in nSMase2. Moreover, the analysis of the residues contributing to the binding identified His463 as a crucial residue. Interestingly, the inhibitory activity of DPTIP on the H463A mutant protein supported our hypothesis. Thus, an allosteric cavity in nSMase2 has been identified that can be exploited for the development of new inhibitors with anti-flaviviral activity.

## 1. Introduction

*Flavivirus* is a genus of enveloped positive-strand RNA viruses from the family *Flaviviridae*, comprising globally emerging and re-emerging pathogens such as Zika virus (ZIKV), Dengue virus (DENV), Yellow Fever virus (YFV), Japanese encephalitis virus (JEV), West Nile virus (WNV), Usutu Virus (USUV), and tick-borne-encephalitis virus (TBEV), among others. These viruses are transmitted by the bites of infected mosquitos or ticks [1]. Due to a variety of factors that include climate warming and globalization of travel and trade, the impact of diseases produced by flaviviruses on human and animal health has increased during the last decades, with an estimation of infections up to 400 million people annually [1]. The severity of the illness in infected individuals range from a flu-like disease and skin rash to haemorrhagic fevers, severe neurological diseases such as meningitis, encephalitis, Guillain-Barré syndrome, and birth defects [2,3]. Although some vaccines are available [2], there is still a lack of an effective antiviral treatment for any flaviviral disease.

Traditionally, antiviral research has mainly focused on the development of direct acting antivirals (DAAs), which target essential virus proteins involved in the replication cycle (i.e., protease, viral polymerase, entry, or fusion inhibitors). However, host-directed antivirals (HDAs) targeting host factors hijacked by the virus to complete its life cycle have recently been explored [4], in particular those related to metabolic pathways including lipid metabolism [5]. This new strategy offers the opportunity for the development of broad-spectrum antivirals expected to show higher barrier to resistance than classical DAAs [6].

Regarding lipid metabolism, sphingomyelinases represent an attractive therapeutic target for the treatment of a wide range of diseases. These enzymes regulate exosome production, inflammation, cell cycle regulation, T-cell recruitment and migration, or apoptosis, thus playing a key role on pathologies such as cancer, cardiovascular or neurological diseases, and mental disorders such as alcohol abuse or depression [7,8,9,10,11]. In the case of flaviviruses, the activity of neutral sphingomyelinase 2 (nSMase2) was first associated with the multiplication of WNV and USUV [12]. Moreover, the involvement of nSMase2 in the life cycle of ZIKV through modulation of extracellular vesicle production has also been established [13,14]. 

Human nSMase2 (encoded by the SMPD3 gene) is a phosphodiesterase responsible for the hydrolysis of sphingomyelin (SM) into ceramide and phosphorylcholine (PC), and is activated by diverse extracellular stimuli such as reactive oxygen species (ROS), proinflammatory factors, or chemotherapeutic agents [15]. nSMase2 comprises two major domains: a membrane N-terminal domain (NTD, a lipid binding domain responsible of the interaction with phosphatidylserine (PS)) and a C-terminal catalytic domain (CAT) that is interrupted by a large insertion (residues 175-339). An intermediate section called the juxtamembrane linker (JX) connects both domains and acts as an intramolecular allosteric activator. The crystal structure of the catalytic domain of human nSMase2 lacking the insertion (∆175-339) has been recently solved, revealing a DNase-I-type folding with the conserved residues (Asn130, Glu364, Asp510, Asn512, Asp607, Asp638, and His639) in its active site corresponding to this superfamily of enzymes [16,17]. More importantly, nSMase2 also contains the so-called DK switch, formed by residues Asp430 and Lys435. These residues are universally conserved among mammalian, yeast, and bacterial neutral sphingomyelinases. Comparison of the bacterial and human nSMase2 structures revealed that the formation of a salt bridge between these two residues is necessary for the enzyme to be catalytically active [16]. 

**Figure 1:**
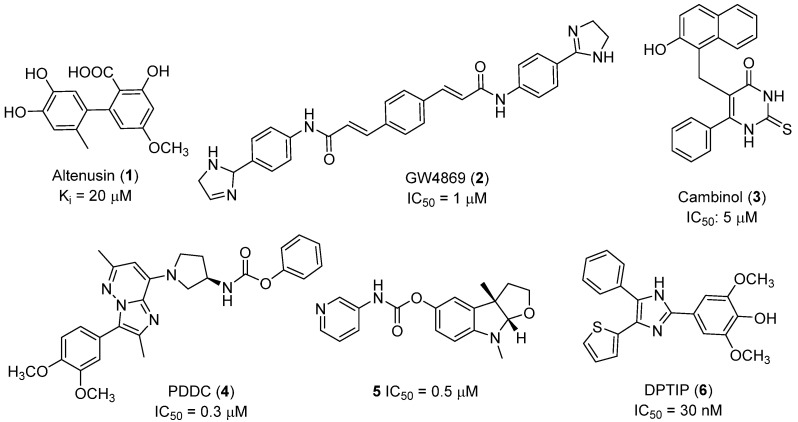
Selected non-competitive inhibitors of human nSmase2.

Recent progress in the development of nSMase2 inhibitors includes a limited number of natural and synthetic compounds with either competitive, non-competitive, or a mixed mechanism of action [18]. The most relevant non-competitive inhibitors are shown in Figure 1. Altenusin (**1**) [19,20], GW4869 (**2**) [21], and Cambinol (**3**) [22,23] have showed low potency against nSMase2 (in the µM range) and present some pharmacokinetics problems such as low solubility and/or metabolic instability. On the other hand, PDDC (**4**) [24], carbamate (**5**), recently described as a dual sphingomyelinase-2 and acetylcholinesterase inhibitor [25], and, specially, DPTIP (**6**) [26] with an IC_50_ value of 30 nM represent the most potent inhibitors reported to date.

Unfortunately, no structural information of the binding site(s) of non-competitive inhibitors of nSMase2 is available, thus hampering a rational approach towards new series of compounds. To the best of our knowledge, only a computational docking study has been reported with Cambinol (**3**) [23], although no experimental data to confirm the proposed binding site was included. 

Based on the reported involvement of nSMase2 in the replication of flavivirus, we hypothesized that nSMase2 inhibitors were worthy of exploration for antiflaviviral activity. Among the known non-competitive nSMase2 inhibitors (Figure 1), we centered our attention on DPTIP as the most suitable for our study based on all the experimental data available including its potency (IC_50_ = 30 nM), overall good reported ADME properties, and the extensive recently reported structure-activity relationship (SAR) studies on DPTIP derivatives [27]. Here, we report on the antiviral activity of the nSMase2 inhibitor DPTIP against WNV and ZIKV. The good data obtained led us to perform a thorough computational study to unravel its potential allosteric binding site(s) in human nSMase2. These studies have been complemented with site-directed mutagenesis studies in nSMase 2 and their impact on the inhibitory effect of DTPIP. Our aim is to identify an allosteric cavity in nSMase2 that can be further exploited for the rational design of new inhibitors with anti-flaviviral activity, opening new possibilities for therapeutic intervention against these pathogens. 

## 2. Results and Discussion

### 2.1. Antiviral Activity of DPTIP

**Figure 2:**
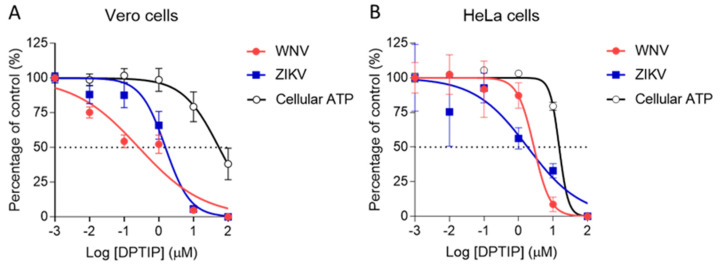
Antiviral activity of DPTIP against medically relevant flaviviruses. Virus yield in the supernatant of Vero (**A**) or Hela (**B**) cells infected with WNV or ZIKV (MOI of 1 PFU/cell) was determined at 24 h post-infection by standard plaque assay as described in Materials and Methods. The cytotoxicity of DPTIP was measured by determination of cellular ATP in uninfected samples. Dashed lines denote a 50% reduction. Data are expressed as mean ± SD (n = 4 to 6).

As a first proof of concept, the antiviral activity of nSMase2 inhibitor DPTIP was tested against WNV and ZIKV as representative medically relevant flaviviruses. To this end, ten-fold serial dilutions of DPTIP were added to infected monolayers of Vero (Figure 2A) or HeLa (Figure 2B) cells and virus yield in the supernatant of infected cultures was determined at 24 h post-infection by plaque assay. Dose-dependent inhibition of virus multiplication was observed for both WNV and ZIKV (Figure 2). Even more, DPTIP showed potent antiviral activity against both viruses with half-maximal effective concentrations (EC_50_) of 0.26 µM in Vero cells and 2.81 µM in HeLa cells for WNV. In the case of ZIKV, EC_50_ values were 1.56 µM in Vero cells and 1.84 µM in HeLa cells. To the best of our knowledge, this is the first time that the antiviral activity of DTPIP against flaviviruses is reported. The good inhibition observed supports that sphingolipid metabolism in the host cell is crucial for virus replication.

The cytotoxicity of DPTIP was estimated in parallel by ATP measurement in uninfected and treated cultures showing a half-maximal cytotoxic concentration (CC_50_) of 54.83 µM for Vero cells and 15.11 µM for HeLa cells which was higher in both cases than EC_50_. Therefore, the calculation of the selective indexes (SI) defined as the ratio of cytotoxicity to antiviral activity (CC_50_/EC_50_) for WNV (206.75 in Vero cells and 5.37 in HeLa) and ZIKV (35.13 in Vero cells and 8.21 in HeLa cells) confirmed the good balance between activity and toxicity pointing to the specificity and safety of the inhibitor.

### 2.2. Modelling of the Missing Loops of nSMase2 Structure and Cavity Probing

**Figure 3:**
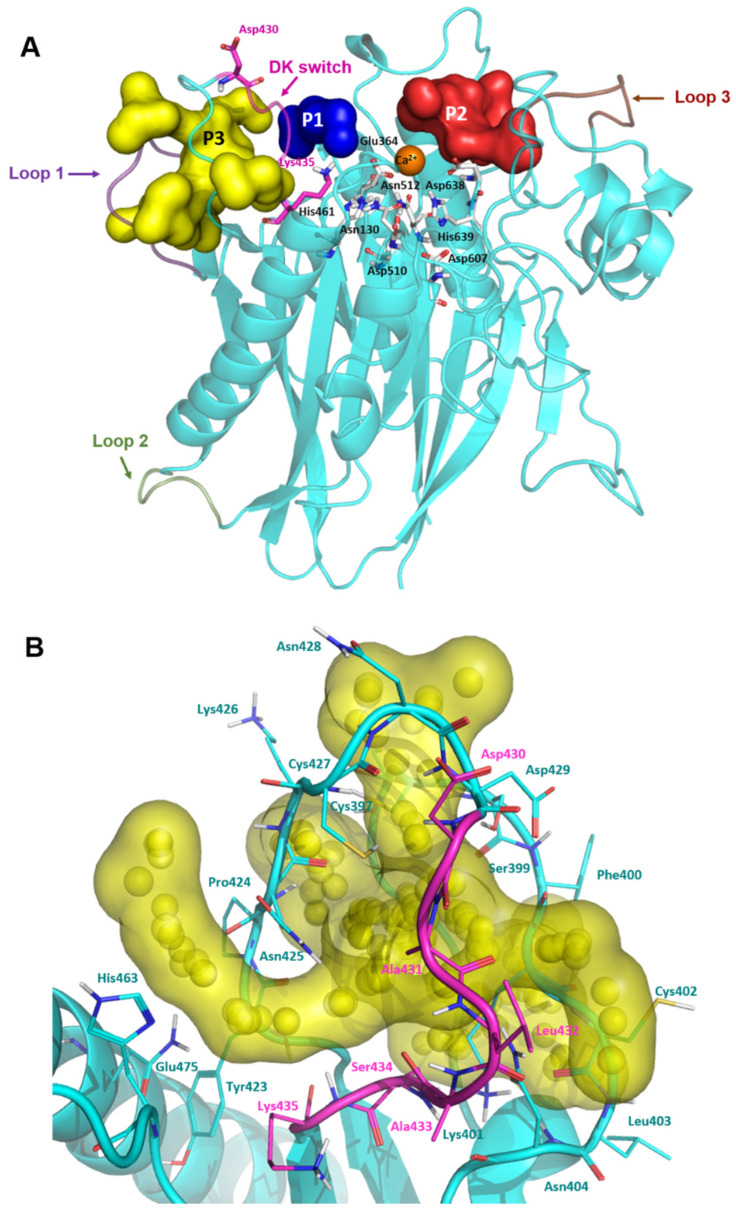
Complete model of human nSMase2 and pocket visualization. (**A**) Modelled loop 1 is shown in violet, loop 2 in green, and loop 3 in brown. DK switch is shown in magenta with residues Asp430 and Lys435 highlighted. Selected pockets are shown in surface and labelled as follows: pocket 1 (P1) in blue, pocket 2 (P2) in red, and pocket 3 (P3) in yellow. Active site residues are shown in grey sticks and labelled and Ca^2+^ is shown as an orange sphere. (**B**) Detailed view of Pocket 3 shown as yellow surface. Spheres represent the alpha spheres calculated by Fpocket. Residues that comprise the pocket are shown in cyan lines and labelled. Residues forming the DK switch are shown in magenta lines and labelled.

Encouraged by the excellent antiviral activity of DPTIP against the flaviviruses tested, we addressed computational studies to unravel the details of its molecular interaction with nSMase2. As mentioned, the reported X-ray structure of human nSMase2 only contains the CAT domain [16], although in this complex there are three loops comprising some regions near the active site that are missing. Loop 1 ranges from residues 393 to 402, also known as the palmitoylation loop, and is located adjacent to the loop containing the DK switch. On the other hand, loop 2, from residues 492 to 498, connects two elements of one of the α/β motifs, while loop 3, from residues 555 to 561, is located opposite to the DK switch and the active site. So, in order to have a more suitable 3D structure of the protein, these missing regions were modelled using the ModLoop server [28] and refined with Prime (Schrodinger Suite) [29] to provide the final model shown in Figure 3A. To validate its stereochemical quality, the modelled structure was examined with Procheck [30]. The Ramachandran plot showed that 99.4% of the residues fell in favoured or allowed regions (see Supplementary Figure S1), supporting the validity of the model.

In order to identify potential binding cavities for allosteric inhibition, this generated model was submitted to the pocket prediction software Fpocket [31,32], resulting in the identification of 27 different cavities. To study the viability of these cavities as potential allosteric sites, they were ranked according to four parameters: the druggability score, the hydrophobicity score, the pocket volume, all of them given by Fpocket, and their overall location in the protein. To select the identified cavities for further analysis, those with a combined relative adequate volume (>300) and a druggability score higher than 0.100 were considered, yielding three possible pockets: pocket 1 (P1), pocket 2 (P2), and pocket 3 (P3) (Table 1 and Figure 3A).

P1 (blue surface in Figure 3A and Figure S2) appeared as a narrow cavity, as expected by the smallest volume of the three selected pockets (Table 1). The high hydrophobicity score in Fpocket of 40, together with available structural data from bacterial nSMases, pointed out that this region could be the hydrophobic groove occupied by the aliphatic chains of SM [16]. Taking into account this information, P1 was discarded.

P2 (represented as red surface in Figure 3A and Figure S2) comprised a larger region adjacent to the active site, with a slightly higher druggability score (Table 1). This region included several residues reported to play a key role in the allosteric activation of the protein by interacting with the juxtamembrane domain (Asn142, Asn143, and Leu144) [17]. It also included the active site residue Asp638. However, this pocket was also discarded due to its low druggability score (Table 1).

P3 (yellow surface in Figure 3A) had the highest volume and druggability score out of the three cavities. Moreover, it included the loop containing the DK switch motif, Asp430, Ala431, Leu432, Ala433, Ser434, and Lys435 (Figure 3B). Other residues worth of note belonging to this pocket were His463 and Gln475, and those belonging to the modelled palmitoylation loop (Gly396, Cys397, Cys398, Ser399, Phe400, Lys401, Cys402, Leu403, and Asn404). As mentioned before, residue Asp430 in the DK switch needs to be reoriented towards Lys435 so that the two residues could be involved in the catalysis. Thus, by blocking this conformational change, nSMase2 activity would be disrupted. Based on this information, P3 was selected as the putative allosteric binding site for this study.

### 2.3. Binding Mode of DPTIP: Docking Studies and Molecular Dynamics Simulations

Extensive structure-activity relationship (SAR) studies on DPTIP derivatives have been recently reported [27]. Some of these SARs are worth to mention since they could shed light on the selection of the putative binding mode(s) of DPTIP. For instance, the presence of the free OH group at position 4 of the phenyl in DPTIP was essential for activity since its removal (as in compound **7**, Table 2) or its replacement by a methoxy group (as in compound **8**) led to completely inactive compounds. It was also relevant the difference in IC_50_ values between DPTIP with a free NH in the imidazole ring and its N-methylated analogue (compound **9**, Table 2), suggesting that the polar NH might play an important role in its binding to the target. Derivatives with bulky groups in the adjacent positions to the OH group (compounds **10** and **11**) showed only a small reduction in inhibitory activity, with IC_50_ values in the low micromolar range (IC_50_ = 0.12 µM and 0.52 µM, respectively.

**Figure 4:**
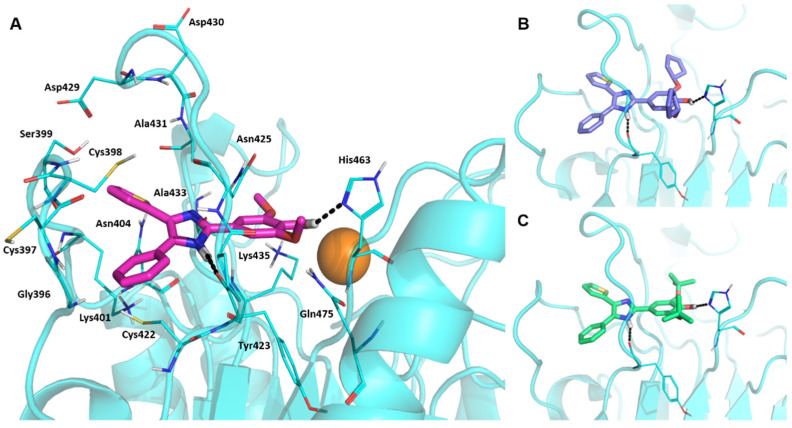
Molecular docking of DPTIP to nSMase2. (**A**) Putative binding mode of DPTIP (magenta sticks) in nSMase2 (shown in cyan) from the docking studies. Selected residues from the binding pocket are shown as cyan lines and labelled. Hydrogen bonds interactions with Tyr423 and His463 are shown as black dashed lines. Docking poses for active DPTIP derivatives 10 (**B**) and 11 (**C**).

Using Autodock [33], DPTIP was submitted to docking in the selected pocket P3. Analysis of the 250 poses provided eight clusters, from which the most energetically favourable conformation of each cluster was studied in detail considering the SARs explained above and prioritising those where hydrogen bonds could be established between residues of the pocket and the OH of the phenol and/or the NH of the imidazole of DPTIP. Under these premises, only one docking pose fulfilled all the requirements and is shown in Figure 4A. In this selected pose, the NH in the imidazole ring of DPTIP formed a hydrogen bond with the backbone carbonyl of Tyr423 with a distance of 2.0 Å whereas the phenolic OH of the ligand is at hydrogen bond distance (2.1 Å) to the Nδ on the imidazole ring of His463. Other residues surrounding the phenolic ring are Lys435, Asn425, and Gln475. On the other side of the DK switch loop, the phenyl ring occupied a region lined by residues as Cys397, Gly396, Cys422, and Lys 401, and the thiophenyl by residues Cys398, Ser399, Asn404, and Ala433. 

Compounds **10** and **11**, which also showed significant inhibition of nSMase2, were submitted to docking studies within the same pocket, resulting in similar interactions to those above described for DPTIP. For instance, compound **10** (Figure 4A) could establish a hydrogen bond between the NH and the backbone carbonyl of Tyr423 (distance: 2.0 Å), and between the phenolic OH and the imidazole ring of His463 (distance 2.1 Å). For compound **11** (Figure 4C), measurements of these distances shed values of 2.5 Å and 2.6 Å. For both compounds, bulky substituents seemed to nicely fit in the cleft where His463 is located.

**Figure 5:**
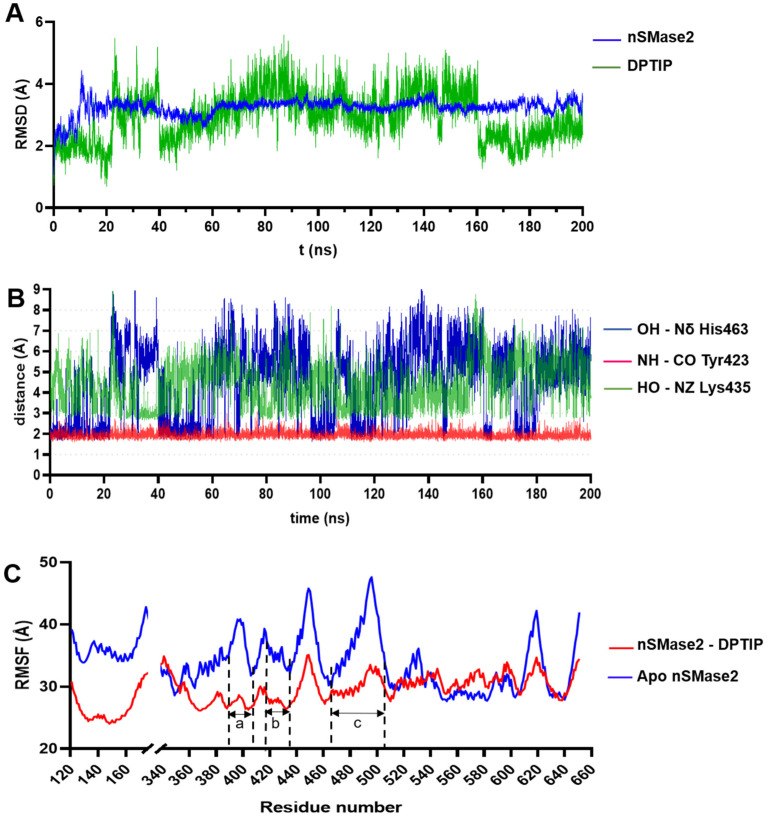
RMSD, critical distances, and RMSF for nSmase2-DPTIP binding based on the MD simulations. (**A**) RMSD plot the nSMase2-DPTIP complex during the MD simulations. (**B**) Evolution of the distance of DPTIP-OH-Nδ-His463, DPTIP-NH-CO Tyr423, and DPTIP-OH-NZ Lys435 along the MD simulation. (**C**) RMSF plot of protein residues during the MD simulations of the nSMase2-DPTIP complex and apo protein.

To study the stability of the DPTIP-nSMase2 complex, molecular dynamics (MD) simulations lasting 200 ns were performed using the Amber16 suite of programs [34]. RMSD of the Cα trace of the protein and of the heavy atoms for DPTIP were monitored along the MD trajectory taking the predicted docking conformation as reference. As shown in Figure 5A, the protein showed an initial stabilization (from 0 to 20 ns) with RMSD values between 2 and 3 Å. Then, after a local rearrangement of the loops, the RMSD values reached a maximum of ~4 Å before being stabilized with RMSD values between 3.4 and 3.6 Å throughout the rest of the simulation. The high amount of loops in the nSMase2 structure, together with their intrinsic mobility, contributed to the high RMSD. On the other hand, DPTIP remained stable within the binding pocket for the first 20 ns of the simulation, with variations in RMSD values of 1 to 2 Å from the reference structure. Although higher RMSD values appeared in the period from 20 to 160 ns, DPTIP reached again the equilibrium for the last 40 ns of the simulation. The nature of the allosteric binding site, formed mainly by two loops, allowed a certain degree of mobility for DPTIP, and could be the reason of the unusual high RMSD values obtained.

The interactions between the polar groups of DPTIP and their putative partners in the protein were monitored though the entire simulation. Thus, the distance between the NH of imidazole ring of DPTIP and the backbone carbonyl of Tyr423 (represented in red, Figure 5B) remained constant along the simulation, reaching a maximum value of 2.8 Å and an average distance of 1.9 ± 0.1 Å. On the other hand, during the first 20 ns of the simulation, the hydrogen bond between the hydroxy group of DPTIP and the Nδ of His463 showed an average distance of 2.4 ± 0.9 Å (shown in blue, Figure 5B) with some periods of larger oscillation, where distances reached values between 4 Å and 6 Å. A detailed visual inspection of the MD trajectory in this period of time showed that a water molecule briefly occupied the space between the phenolic OH group and imidazole ring of His463, getting involved in the hydrogen bond interaction (see Figure S3A) and explaining these higher distance values. In addition, the rest of the MD simulations showed even larger oscillations in the distance between DPTIP and His463. Again, detailed inspection of the MD trajectory led us to identify residue Lys465 as an important actor in the binding of DPTIP. Thus, plotting of the distance between the NZ atom of the side chain of Lys465 and the OH of DPTIP (green line in Figure 5B) showed that this new interaction appeared when His463 was at large distance of DPTIP, as clearly seen comparing the lines during the period of 20 to 40 ns (see also Figure S3B). This behaviour was repeated in the rest of the simulation (Figure S3C). Therefore, in addition to some transient water molecules, the main interactions of DPTIP in the binding sites fluctuated between residues His463 and Lys435.

The nSMase2 model without DPTIP (apo form) was also submitted to MD simulations, following the same protocol as for the DPTIP-nSMase2 complex. This was meant to study the fluctuations of each residue along the simulation and to determine the effect of DPTIP in stabilising the binding pocket area. Comparison of the RMSF values between the apo form and the complex showed lower values for the complex in three important regions: from residues 386 to 406, 417 to 438, and 461 to 500 (Figure 5C). These regions correspond to the palmytoilation loop (region a in Figure 5C), adjacent to the DK switch, the DK switch loop (region b), and the neighbouring residues to His463 (region c), respectively, all surrounding DPTIP in the proposed binding mode within nSMase2. Altogether, the binding of DPTIP resulted in a stabilisation of the loops that shaped the identified pocket. 

**Figure 6:**
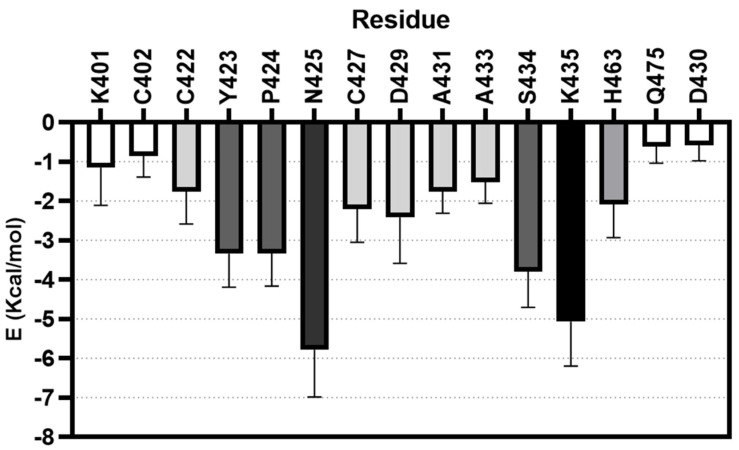
Energy contribution per residue given by MM_ISMSA for the complex nSmase2-DPTIP.

In addition, the MM-ISMSA software [35,36] was used to determine the contribution of the individual residues to the free energy of the binding of DPTIP along the MD simulations (Figure 6), being specially relevant the contribution of Asn425, Lys435, Ser434, Tyr423, and Pro424, along with Asp429 and His463. The total estimated free binding energy was −43 ± 3 Kcal/mol. 

In order to confirm the proposed binding mode of DPTIP, we envisioned a computational site-directed mutagenesis (alanine scan) to find the best candidate for the expression of a mutant nSMase2 for which DPTIP binding could be affected. From the results obtained from the energy analysis and the MD simulation, the feasible candidates identified were Asn425, Lys435, Ser434, Tyr423, Pro424, Asp429, and His463.

Among these residues, Lys435 was promptly discarded due to its role in the activation of the catalytic form of nSMase2 as part of the DK switch. On the other hand, the main component of the interaction of Asn425, Ser434, Pro424, and Asp429 with DPTIP was of hydrophobic nature (Van der Waals), so they were not considered as suitable candidates either. Focusing on the disruption of polar interactions, Tyr423 seemed adequate. However, Tyr423 was discarded as the interaction with DPTIP was established through its backbone carbonyl. Finally, His463 was selected as the most suitable candidate for alanine replacement considering the polar interaction between its side chain and DPTIP.

The H463A mutant was modelled and refined with Prime following the same protocol as with nSMase2 wild type (WT). Then, docking studies with DPTIP were carried out. As expected, the analysis of the results indicated that the putative binding pose of DPTIP in the nSMase2 WT was not present in the H463A mutant protein, thus further pointing to the key contribution of His463 to the binding mode. Even though the proposed pose for DPTIP with nSMase2 was not present with the H463A mutant, DPTIP was manually docked into the H463A nSMase2 model and submitted to MD simulations under the same protocol as the WT nSMase2-DPTIP complex model, and similar parameters were monitored for its comparison.

**Figure 7:**
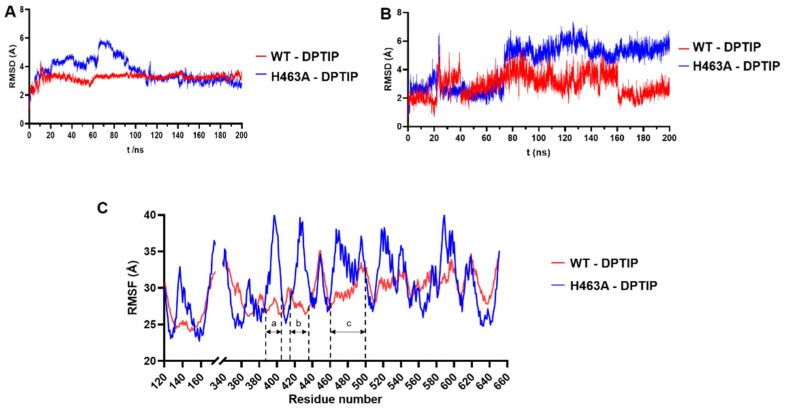
Analysis of the MD simulations of nSMase2 mutant H463A on DPTIP binding. (**A**) RMSD plot of WT-DPTIP and H463A-DPTIP complexes during the MD simulation in apo form. (**B**) RSMD plot of DPTIP in the complex with WT nSMase2 and the H463A mutant. (**C**) RMSF per residue (the PDB includes a deletion between residues 175 and 339 and for clarity purposes the residue numbering is maintained and the gap in the graph represents the mentioned deletion).

As shown in Figure 7A, RMSD values of the Cα trace of the WT and H463A proteins showed in general similar tendencies. However, more relevant differences were observed in the RMSD calculated for DPTIP in both complexes (Figure 7B), where DPTIP RMSD values were higher in the H463A complex than those of the WT-DPTIP complex, pointing to a weaker stabilization of DPTIP inside the H463A complex. The same tendency was observed in the RMSF curves, where values for H463A-DPTIP complex (represented in blue in Figure 7C) were higher than those for the WT complex, especially in the putative binding site region, involving residues 388-408 (palmitoylation loop, region a in Figure 7C), 415-435 (DK-switch loop, region b in Figure 7C), and with a minor, but still significant, difference from residues 463-482 (region c in Figure 7C).

More importantly, the total estimated binding energy for the H463A-DPTIP complex was −39 ± 4 Kcal/mol while that of the WT nSMase2-DPTP complex was −43 ± 4 Kcal/mol. Altogether, including the difference in the binding free energy of 5 kcal/mol, the results obtained pointed to the important role of His463 in the binding of DPTIP to nSMase2 and this residue was selected for the expression of a mutant nSMase2.

### 2.4. nSMase2 Inhibition Assay

**Figure 8:**
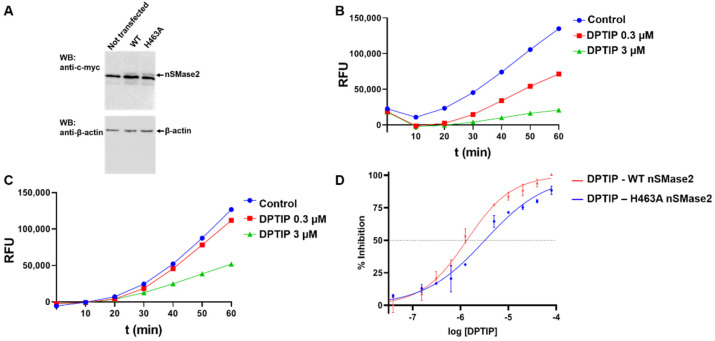
Inhibition of WT and H463A nSMase2 by DPTIP. (**A**) Expression of recombinant nSMase2. Plasmids encoding WT or H463 mutant were transfected into 293T cells and the expression of recombinant nSMase2 variants was determined by western blot using an anti-c-myc antibody 48 h post-transfection. Not transfected cell lysate were included as a negative control. β-actin was detected as a control for protein loading. (**B**,**C**) Fluorescence intensity curves at two DPTIP concentrations for WT nSMase2 (**B**) and for H463A nSMase2 (n = 3). RFU denotes random fluorescence units. (**D**) IC_50_ curves of DPTIP with WT and H463A nSMase2 cell lysates (n = 2).

WT and H463A recombinant nSMase2 were produced in 293T cells (Figure 8A). nSMase2 activity was measured by monitoring resorufin fluorescence, employing the Amplex™ Red reagent coupled with enzymes choline oxidase, alkaline phosphatase, and horseradish peroxidase as previously described [22]. Firstly, fluorescence intensity (FI) response was adjusted for WT and H463A cell lysates to determine the total protein concentration needed to obtain a similar level of activity. Final total protein concentrations were set at 2.5 µg/mL for WT nSMase2 and 7.5 µg/mL for H463A nSMase cell lysates. Then, an assay monitoring FI was performed to see if fluorescence intensity curves showed a different pattern for WT and H463A with the same concentration of DPTIP after 1h of incubation (Figure 8B,C). The higher the inhibition, the less phosphorylcholine is produced, which in turn leads to less resorufin generation and a lower fluorescence signal. When comparing the intensity of the fluorescence signal for DPTIP at a fixed concentration (3 and 0.3 uM) in WT cell lysates (Figure 8B) and in H463A cell lysates (Figure 8C), it became clear that the intensities at both concentrations are higher for the mutant than for the WT, thus indicating a lower level of inhibition of the mutant in the presence of DPTIP. In addition, concentration-response curves were obtained to determine IC_50_ values (Figure 8D). In our hands, DPTIP showed an IC_50_ value of 1.35 µM against WT nSMase2 and 3.22 µM against H463A mutant. The higher IC_50_ value obtained for the mutant H463A points to the importance of this residue in the binding of DPTIP to nSMase2. Still, as shown in the MD simulations, both the backbone CO of Tyr423 and the NH_2_ group of the side chain of Lys465 significantly contribute to the binding of DPTIP and this could account for the relative inhibition obtained in the cell lysates of the mutant H463A.

## 3. Materials and Methods

### 3.1. DPTIP

DPTIP was synthesized as described in the literature [26]. ^1^H NMR (300 MHz, d_6_-DMSO): δ 3.85 (6H, s), 6.97 (1H, dd, J = 3.6 Hz, 5.1 Hz), 7.04 (1H, d, J = 3.0 Hz), 7.33 (2H, s), 7.36–7.44 (2H, m), 7.51 (2H, t, J = 7.6 Hz), 7.59 (2H, d, J = 7.0 Hz), 8.65 (1H, s), 12.54 (1H, s). ^13^C-NMR (300 MHz, d_6_-DMSO): δ 56.65, 103.63, 120.95, 122.98, 124.48, 127.55, 128.99, 131.64, 132.15, 136.89, 138.87, 146.49, 148.67. MS (ES, positive mode): 379 [M+H]+. See Supplementary Figure S4.

### 3.2. Virus Infections and Drug Treatments

Infectious virus manipulations were performed in BSL-3 facilities on Vero cells (ATTC CCL-81). The origin of WNV New York 99 and American ZIKV PA259459 has been previously described [37]. Infections in liquid medium and virus titrations in semisolid agar medium were performed as described [38,39]. Briefly, to determine the antiviral activity of DPTIP, Vero cells cultured in Minimum Essential Medium Eagle (MEM, Corning, NY, USA) were infected with WNV or ZIKV at a multiplicity of infection (MOI) of 1 plaque forming unit (PFU)/cell. After 1 h of adsorption at 37 °C, viral inoculum was removed, and fresh medium supplemented with 1% fetal bovine serum and containing DPTIP was added. Virus titers were determined 24 h post-infection by standard plaque assay in semisolid agar medium. The cytotoxicity of DPTIP was determined in parallel in uninfected cultures by quantification of cellular ATP using Cell Titer Glo luminescent cell viability assay (Promega, Madison, WI, USA).

### 3.3. Production of Recombinant nSMase2

Recombinant nSMase2 (SMPD3) was produced using an expression plasmid that encodes the cDNA of human NSMase2 (SMPD3) (NM_018667) fused to Myc-DDk tag cloned between SgfI-MluI restricition sites of pCMV6-Entry vector (OriGene technologies, Inc., Rockville, MD, USA, Catalog # RC218441). Plasmid encoding mutant nSMAse2 with amino acid replacement histidine 463 to alanine (H463A) was produced by directed mutagenesis with NZYMutagenesis kit (nzytech) and mutagenic oligonucleotide primers cctgcacacacctg**gct**gccccacaagagg and cctcttgtggggc**agc**caggtgtgtgcagg. Mutated nucleotides are indicated in bold. The nucleotide identity of the plasmids was verified by automated nucleotide sequencing (Marcogen Spain Inc., Madrid, Spain). Plasmids were amplified in *E. coli* and purified with Purelink HiPure Plasmid Filter Maxiprep Kit (Invitrogen, MA, USA). For recombinant protein expression, plasmids were transfected into human embryonic kidney (HEK) 293-T cells (ATCC CRL-11268) with DharmaFECT kb DNA transfection reagent (Horizon Discovery Ltd., Cambridge, UK). Cells were harvested at 48 h post-transfection with a cell scraper, resuspended in 100 mM Tris-HCl (pH 7.4), 10 mM MgCl_2_ lysis buffer, and sonicated for 30 s 3 times. The amount of protein in cell lysates was determined by Bradford assay and the expression of recombinant protein was verified by Western blot [40] using anti-c-myc clone 9E10 (ATCC), mouse monoclonal anti-β-actin AC-15 (Sigma, Kawasaki, Japan), and anti-mouse IgG coupled to horseradish peroxidase as secondary antibody (Sigma).

### 3.4. nSMase2 3D Model: Modelling, Docking and Molecular Dynamics Simulations

#### 3.4.1. Protein Model

The crystal structure of nSMase2 with a deleted insertion (amino acids 175-339) was retrieved from the Protein Data Bank (PDB ID: 5UVG). This model corresponds only to the C-terminal or catalytic domain [16,41]. The missing loops close to the active site were modelled using the ModLoop webserver [28] and refined using the Prime module of Schrödinger software suite [29,42]. The quality of the whole protein model was validated with Procheck [30]. The protonation states for the protein residues were studied with AMBER force field [43]. 

#### 3.4.2. Binding Site Prediction

From the final nSMase2 model, possible binding sites were screened using the open-source pocket detection software Fpocket (available at https://fpocket.sourceforge.net/; accessed on 20 October 2022). Based on Voronoi tessellation and alpha-spheres [31,32]. The software core workflow is organized in three steps: (i) the whole ensemble of alpha spheres, a sphere that contacts four atoms on its boundary and contains no internal atom, is determined from the protein structure; (ii) identification of clusters of spheres close together, identification of pockets, and removing clusters of poor interest; and (iii) calculation of properties from the atoms included in the pocket to score each pocket.

#### 3.4.3. Docking

Docking studies were carried out using AutoDock 4.2 [33]. Preparation of nSMase2, DPTIP, and derivatives **10** and **11** pdbqt files were performed with AutoDock Tools 1.5.6. A three-dimensional cubic grid, consisting of 45 × 45 × 45 points with a spacing of 0.375 Å, was defined at pocket P3, which includes residues from the palmitoylation loop, the loop containing the DK switch motif, along with His463 and Gln475. Electrostatic, desolvation, and affinity maps for the atom types present in DPTIP, 10, and 11 were calculated using AutoGrid 4.2.6. The Lamarkian genetic algorithm implemented in AutoDock 4.2 was used to generate the docked conformations within the putative binding cavity by randomly changing the overall orientation of the molecule as well as the torsion angles of all rotatable bonds. Default settings were used except for the number of runs, population size, and maximum number of energy evaluations, which were fixed at 250, 100, and 250,000, respectively. Rapid intra- and intermolecular energy evaluations of each configuration was achieved by having the receptor’s atomic affinity potentials for aliphatic and aromatic carbon, oxygen, nitrogen, and hydrogen atoms pre-calculated in the three-dimensional grid. A distance-dependent dielectric function was used in the computation of electrostatic interactions. The 250 poses obtained were analysed, clustered, and ranked based on the calculated binding energies given by Autodock. The best conformation of each of the eight clusters obtained were visually inspected to study the putative interactions within the binding site.

#### 3.4.4. Molecular Dynamics Simulations

The selected pose from the docking study was used as a starting point for the MD simulations and were carried out using the Amber16 suite of programs [34]. The ff14SB force field [43] was used for the protein in combination with the TIP3P water model [44], and the GAFF2 [45] for the parametrization of the ligand. 

The molecular systems consisting of DPTIP, Ca^2+^ ion, and nSMase2 or H463A mutant were neutralized by the addition of 12 Na^+^ ions [46] and immersed in a truncated octahedron of 10,000 TIP3P water molecules. Periodic boundary conditions were applied and electrostatic interactions were treated using the smooth particle mesh Ewald (PME) method [47] with a grid spacing of 1 Å. The cutoff distance for the non–bonded interactions was 9 Å. The SHAKE algorithm [48] was applied to all bonds and an integration step of 2.0 fs was used throughout. Solvent molecules and counterions were relaxed by energy minimization and allowed to redistribute around the positionally restrained solute (5 kcal·mol^–1^·Å^–2^) during 50 ps of MD at constant temperature (300 K) and pressure (1 atm). These initial harmonic restraints were gradually reduced in a series of progressive energy minimizations until they were completely removed. The resulting systems were heated again from 100 to 300 K during 20 ps and allowed to equilibrate in the absence of any restraints for 200 ns during which system coordinates were collected every 2 ps for further analysis.

Three–dimensional complexes structures and trajectories were visually inspected using computer graphics program PyMOL [49]. Interatomic distances, root–mean–square deviations (RMSD), and root-mean-square fluctuations (RMSF) from a given structure were monitored using the *cpptraj* [50] module in AMBER. Data analysis and plotting was performed with GraphPad Prism 8. Free binding energies and energy contributions per residue between DPTIP, nSMase2, and H463A mutant were calculated employing the MM_ISMSA method [35,36]. 

### 3.5. Fluorescence-Based nSMase2 Activity Assay

nSMase2 activity was measured using a fluorescence-based assay. All the reagents necessary for the fluorescence measurement were included in the Amplex™ Red Sphingomyelinase Kit (ThermoFisher Scientific, Waltham, MA, USA). The assay was carried out in black solid bottom, 96-well plates (ThermoFisher, Nunc™ F96), with a final volume of 200 µL, using the Amplex Red^®^ reagent as described before [22]. In a two-fold decreasing manner, concentrations ranging from 20 to 0.078 µg/mL of recombinant human nSMase2 cell lysate were incubated for 30 min. For the single-point mutant H463A, concentrations ranging from 40 to 0.313 µg/mL were measured. Fluorescence intensity (FI) was measured by monitoring the transformation of Amplex Red^®^ into resorufin (ex/em 530/590 nm). To the solutions containing the protein and DPTIP, a solution containing Amplex Red^®^ (50 µM), SM (0.25 mM), and coupling enzymes (Alkaline phosphatase 4 U/mL, Choline oxidase 0.1 U/mL, Horseradish peroxidase 1 U/mL) in reaction buffer (0.1 M Tris-HCl, 10 mM MgCl_2_, pH 7.4, 0.2% Triton X-100) was added. FI was determined with a CLARIOstar^®^ microplate reader (BMG Labtech, Ortenberg, Germany) at 37 °C. Cell lysates were preincubated at 37 °C for 15 min prior to the addition of the fluorescent solution, and then incubated for an additional 1h. Generation of resorufin was monitored by measuring relative fluorescent units (RFU). 

The percentage of inhibition was calculated relative to the intensity of the control in the absence of inhibitor. The change in FI of the sample of inhibitor was divided by the change of FI of the control, giving the relative activity. Subtraction of relative activity to 100 provided the relative percentage of inhibition. Percentage of inhibition for the IC_50_ calculation was expressed as the average of two individual experiments run in triplicate.

### 3.6. Data Analysis

Antiviral activity and cytotoxicity data are presented as mean ± standard deviation (SD). Moreover, 95% confidence intervals (CI) of the mean were also calculated. The number of independent biological replicates (n) analyzed is indicated in the figure legends. Non-linear curve fitting was performed with Prism 7 or 8 for Windows (GraphPad Sofware, San Diego, CA, USA). Dose-response curves were calculated by adjusting the sigmoidal log (inhibitor) vs normalized response (variable slope).

## 4. Conclusions

In summary, the antiviral activity of the nSMase2 inhibitor DPTIP against the flaviviruses WNV and ZIKV has been reported here for the first time. The potent antiviral activity observed, with EC_50_ values within or below the low µM range for WNV and ZIKV, respectively, agree with previous observations pointing that the sphingolipid metabolism in the host cell is crucial for virus replication. Based on these data, we have carried out an intensive in silico study to propose the binding mode of DPTIP in an unexplored allosteric pocket in nSMase2. Taking in to account the SAR data reported for nSMase2 inhibition of DPTIP analogues, the resulting docking poses were narrowed down to one involving hydrogen bonds interactions between the phenolic OH of DPTIP and the side chain of His463 and between the NH on the imidazole ring of DPTIP and the backbone carbonyl group of Tyr 423. Molecular dynamics simulations further stressed the important role of Lys465 and H463 in the interaction between DPTIP and nSMase2, that were further supported by computational studies performed with the mutant H463A protein. The inhibition of DPTIP was compared in a fluorescence assay using WT nSMase2 and mutant H463A in cell lysates. The reduction of the inhibition obtained against the H463A nSMase2 supported the computational data. Thus, an allosteric binding site where DPTIP can be lodged has been identified, cavity that can be further explored for the rational design of new chemical classes of allosteric nSMase2 inhibitors. Altogether, our findings pave the way for new antivirals against flavivirus by targeting host sphingomyelin metabolism thorough inhibition of nSMase 2. 

## Figures and Tables

**Table 1:** Data from the Fpocket prediction for the selected putative binding sites.

Pocket.	Volume	Druggability Score	Hydrophobicity Score	Location
**1**	321	0.104	40	Adjacent to active site and to DK switch
**2**	1000	0.168	29	Adjacent to active site
**3**	1618	0.820	22	Adjacent to active site. Includes DK switch

**Table 2:** Data from Fpocket prediction for the selected putative binding sites.

Compound	Structure	IC_50_ (µM)
DPTIP (**6**)	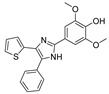	0.03 ^a^
**7**	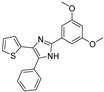	>100 ^a^
**8**	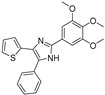	>100 ^a^
**9**	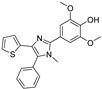	40 ^a^
**10**	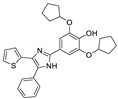	0.12 ^a^
**11**	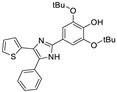	0.52 ^a^

^a^ IC_50_ values reported in [27].

## Supplementary Materials

The supporting information can be downloaded at https://www.mdpi.com/article/10.3390/ijms232213935/s1.
