# Supplementary material for: Allosteric Inhibition of Neutral Sphingomyelinase 2 (nSMase2) by DPTIP: From Antiflaviviral Activity to Deciphering Its Binding Site through In Silico Studies and Experimental Validation
Source: Int J Mol Sci. 2022 Nov 11;23(22):13935. doi: 10.3390/ijms232213935 (PMC9697135; doi:10.3390/ijms232213935)

Supplementary material related to the article:

Allosteric inhibition of neutral sphingomyelinase 2 (nSMase2) by DPTIP: from antiflaviviral activity to deciphering its binding site through in silico studies and experimental validation

Hadrián Álvarez-Fernández,[1] Patricio Mingo-Casas,[2] Ana-Belén Blázquez,[2] Flavia Caridi,[2] Juan Carlos Saiz[2], María-Jesús Pérez-Pérez[1], Miguel A. Martín-Acebes[2] and Eva-María Priego[1*]

1 Instituto de Química Médica (IQM), CSIC,28006 Madrid, Spain

2 Department of Biotechnology, Instituto Nacional de Investigación y Tecnología Agraria y Alimentaria (INIA), CSIC, 28040, Madrid, Spain

Includes:

Figure S1**.** Ramachandran plot of nSMase2 model S2

Figure S2. Detail view of pockets P1 and P2 S3

Figure S3**.** Superimposition of selected MD simulation points S4

Figure S4**.** ^{1}H-RMN, ^{13}C-RMN spectra and HPLC chromatogram of DPTIP S5

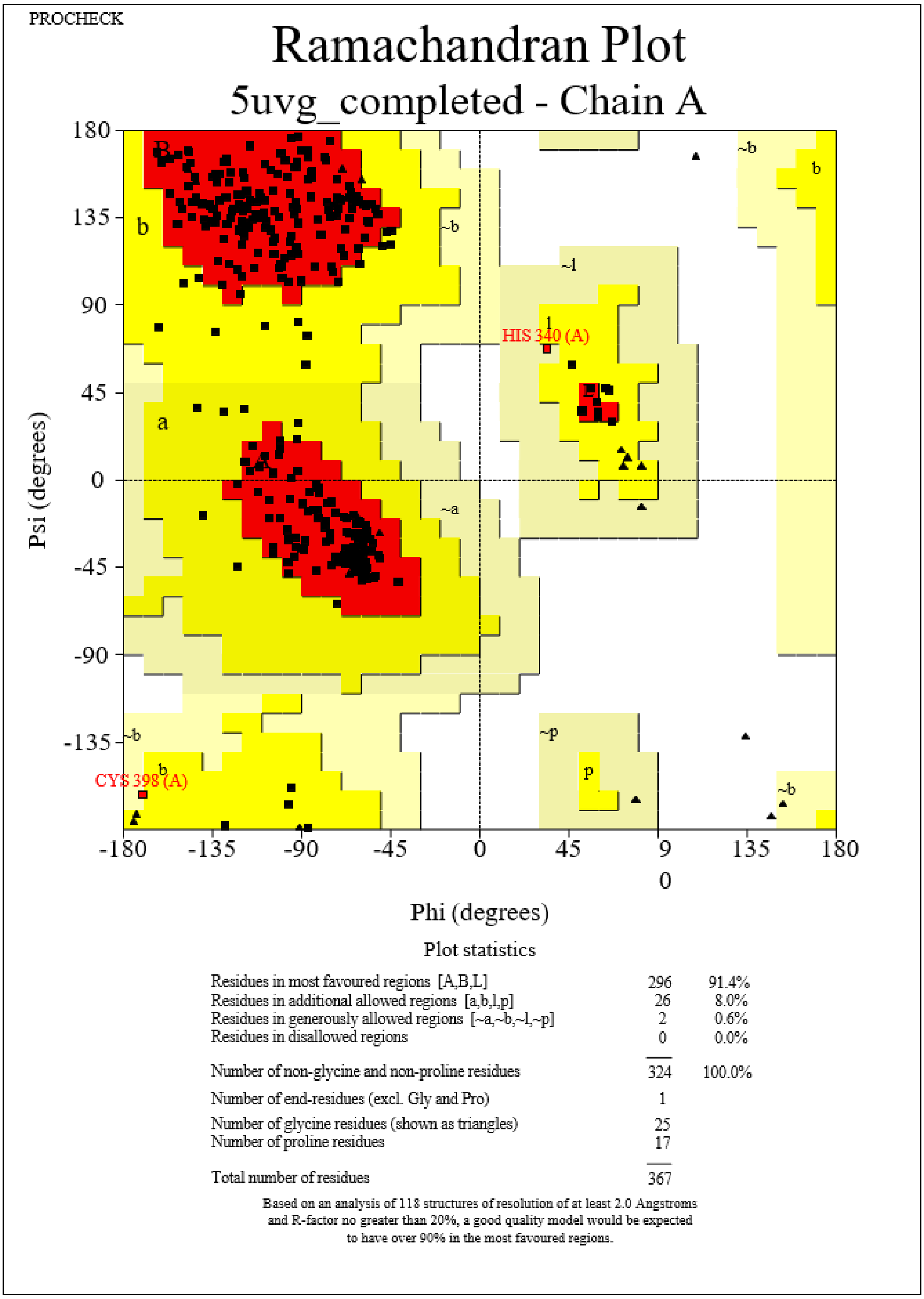

Figure S1. Ramachandran plot of nSMase2 model after loop modeling and structure refinement.

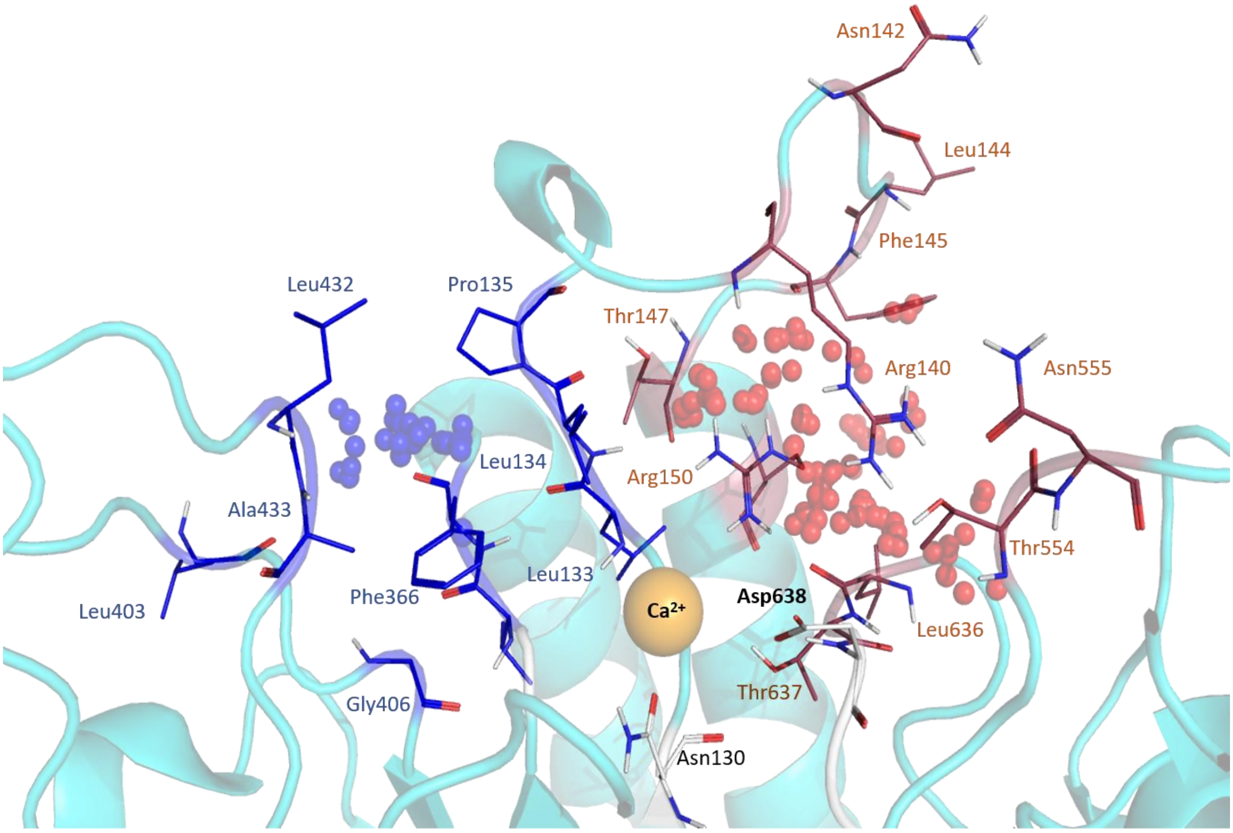

Figure S2. P1 (alpha spheres from Fpocket shown in blue and residues in blue lines) and P2 (alpha spheres shown in red spheres and residues in red lines) Active site residues are shown in grey lines and labeled

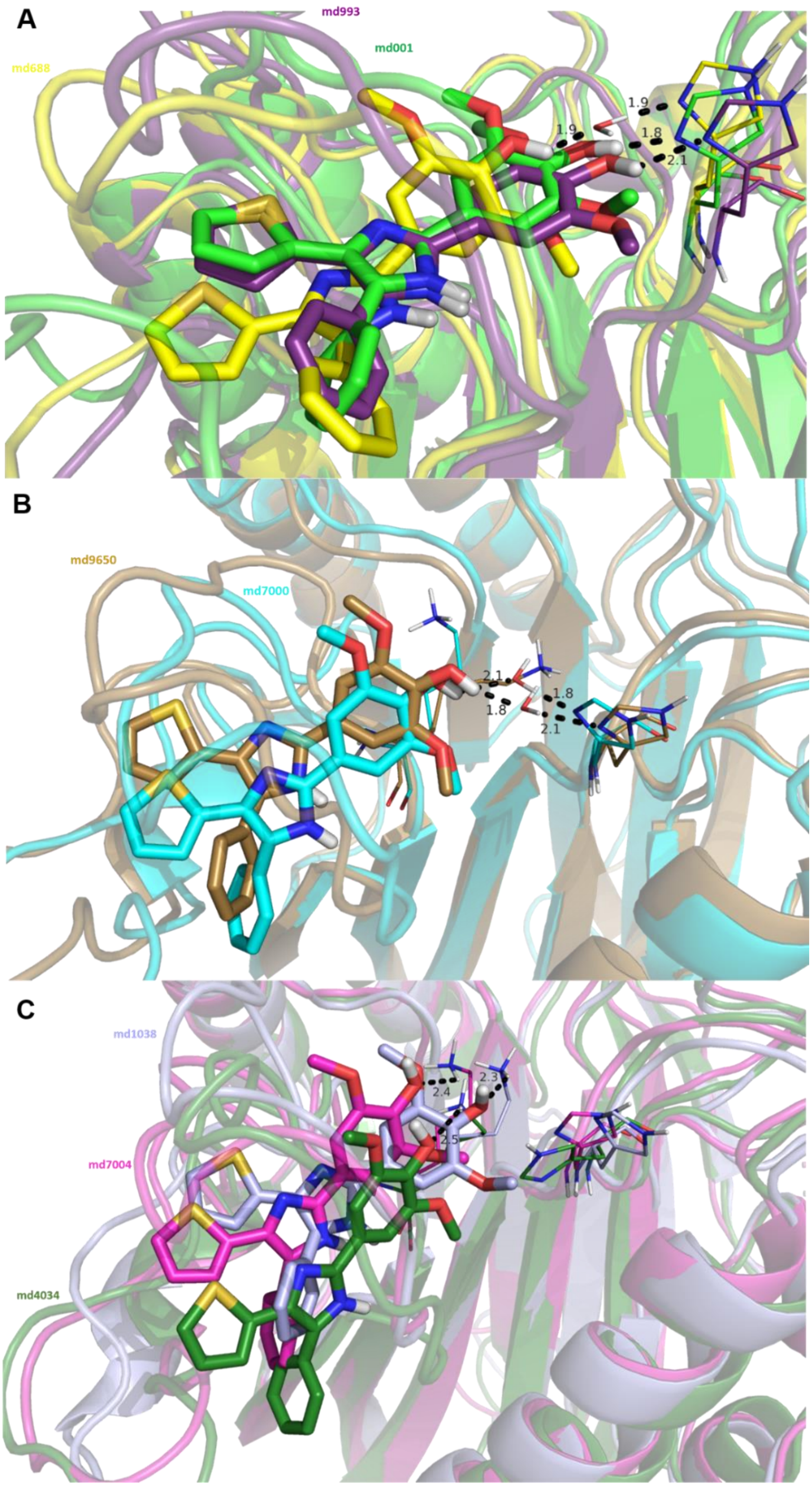

Figure S3. (A) and (B) Superimposition of selected MD simulation points showing the DPTIP phenolic OH – Nδ His463 interaction.(A) Md 001 (0 ns, green), md688 (13.76 ns, yellow) and md993 (19.86 ns, purple); (B) Md7000 (140 ns, cyan) and Md9650 (193 ns, sand). (C) Superimposition of selected MD simulation points showing the DPTIP phenolic OH – Nz Lys465 interaction. Md1038 (20,76 ns, lightblue), md4034 (80,68 ns, green) and md7004 (140,08 ns, magneta). Hydrogen bonds are shown as dashed lines and distances are indicated in Å. Water molecules are shown in lines.

Figure S4. ^{1}H-RMN, ^{13}C-RMN spectra and HPLC chromatogram of DPTIP

(A) ^{1}H-RMN spectrum of DPTIP

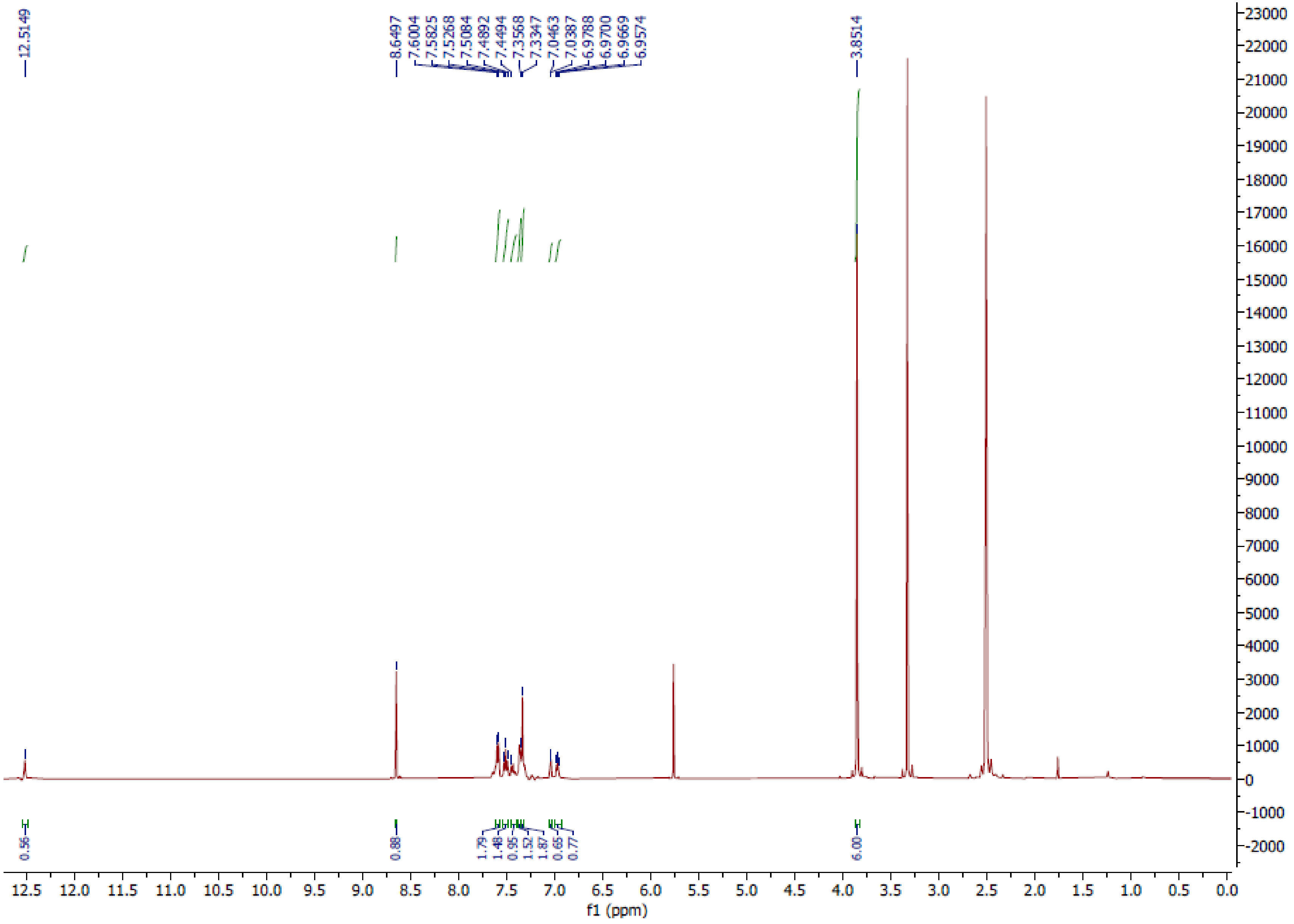

(B) ^{13}C-RMN spectrum of DPTIP

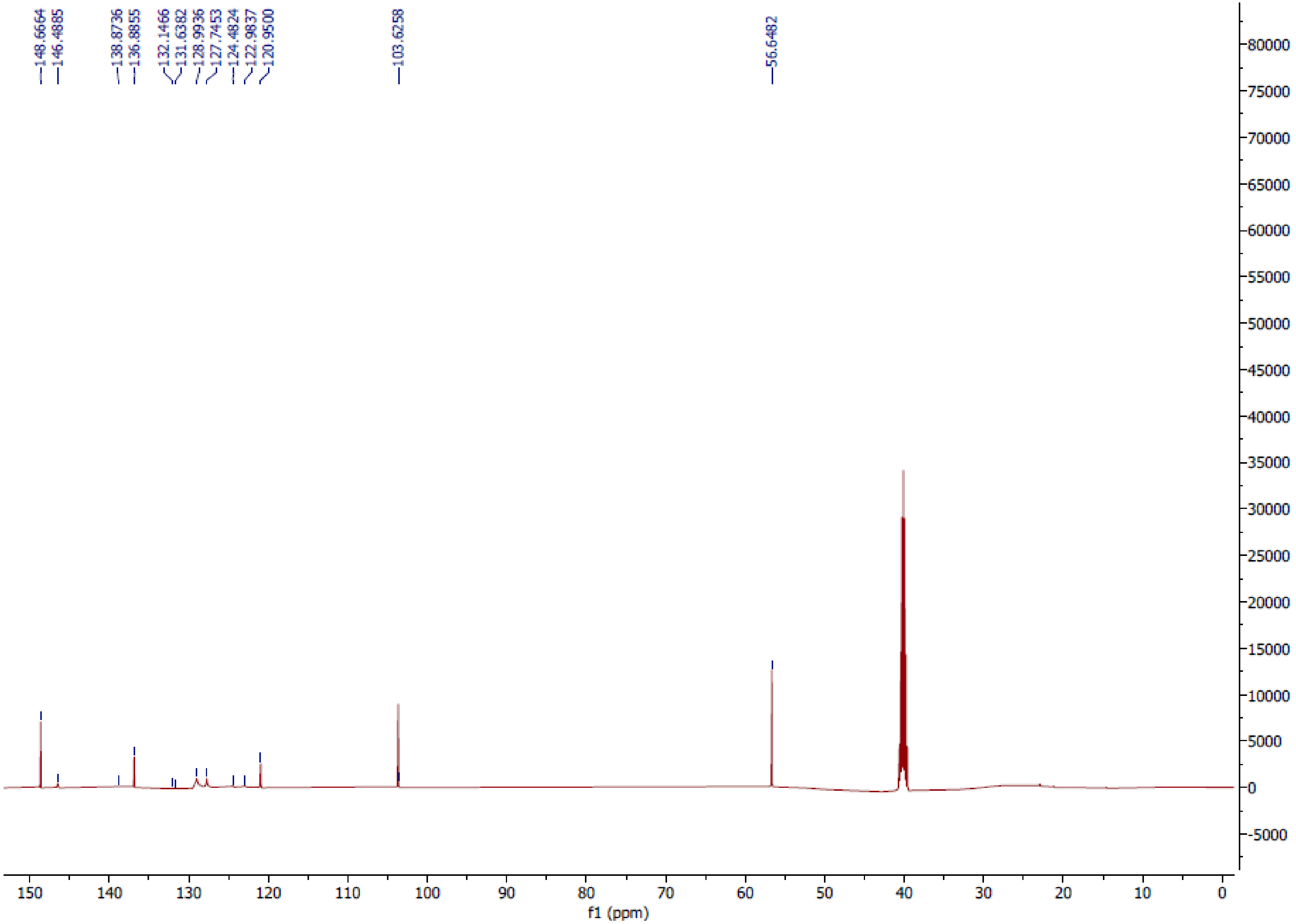

(C) HPLC Chromatogram of DPTIP (Rt = 6.7 min) at λ 254 nm.

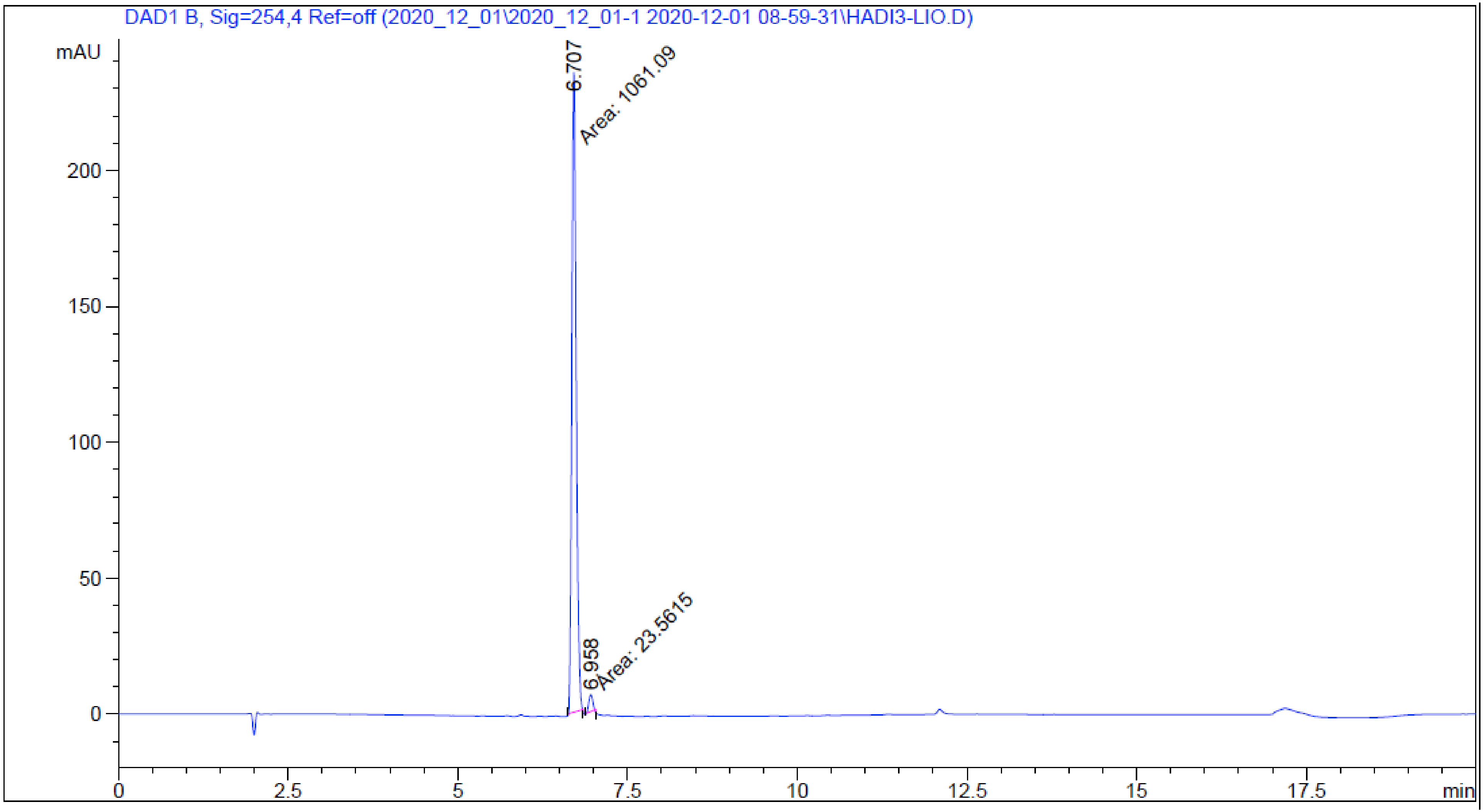

Supplement: Supplementary file 1 [file ijms-23-13935-s001.zip › ijms-1935711-supplementary.pdf]
